# Conspicuous stripes on prey capture attention and reduce attacks by foraging jumping spiders

**DOI:** 10.1098/rsos.230907

**Published:** 2023-11-22

**Authors:** Lauren Gawel, Erin C. Powell, Michelle Brock, Lisa A. Taylor

**Affiliations:** ^1^ Entomology and Nematology Department, University of Florida, 1881 Natural Area Drive, Gainesville, FL 32611, USA; ^2^ Florida State Collection of Arthropods, Florida Department of Agriculture and Consumer Services, Division of Plant Industry, 1911 SW 34th St, Gainesville, FL 32608, USA

**Keywords:** salticidae, achromatic contrast, aposematism, predator–prey interactions, warning colours, warning patterns

## Abstract

Many animals avoid predation using aposematic displays that pair toxic/dangerous defences with conspicuous achromatic warning patterns, such as high-contrast stripes. To understand how these prey defences work, we need to understand the decision-making of visual predators. Here we gave two species of jumping spiders (*Phidippus regius* and *Habronattus trimaculatus*) choice tests using live termites that had their back patterns manipulated using paper capes (solid white, solid black, striped). For *P. regius,* black and striped termites were quicker to capture attention. Yet despite this increased attention, striped termites were attacked at lower rates than either white or black. This suggests that the termite's contrast with the background elicits attention, but the internal striped body patterning reduces attacks. Results from tests with *H. trimaculatus* were qualitatively similar but did not meet the threshold for statistical significance. Additional exploratory analyses suggest that attention to and aversion to stripes is at least partially innate and provide further insight into how decision-making played out during trials. Because of their rich diversity (over 6500 species) that includes variation in natural history, toxin susceptibility and degree of colour vision, jumping spiders are well suited to test broad generalizations about how and why aposematic displays work.

## 1. Introduction

A well-documented strategy for avoiding predators is for prey to defend themselves with chemicals or other defences and then to advertise these defences to potential predators using conspicuous colours and patterns (reviewed in [1]). Across animals, such aposematic displays have typical features that often involve long-wavelength colours (e.g. reds and oranges), bold and contrasting patterns (e.g. black and white stripes), or sometimes elements of each of these strategies and, as such, many animals have evolved innate avoidance of such colour schemes (reviewed in [2]). Foraging animals also often learn to avoid these conspicuous colour schemes more quickly than non-conspicuous alternatives [3–6]. Whether these avoidance patterns are innate or learned, the result is that visually hunting predators are typically highly attentive to these characteristic colours and patterns in prey and avoid attacking them.

While many aposematic displays share similar features as described above, they are also quite diverse [1]. One way to understand how this diversity has evolved is to examine the decision-making of the predators that have driven their evolution [7–9]. This ‘predator psychology' approach was embraced by researchers studying the foraging behaviour of insectivorous birds in the 1990s and research on birds still continues to dominate this field today, which has led to a rich theoretical literature on the nature of aposematic signals [1,10–12]. There has been growing acknowledgement of the importance of expanding this work to include more diverse suites of predators [13] and including non-avian predators in the study of aposematism (e.g. dragonflies [14], mantids [15], bats [16], spiders [17], lizards [18]). Despite this positive trend, the many recent reviews on topics surrounding aposematism highlight how little we know about predators from certain taxa [19–25]). Particularly understudied are the terrestrial invertebrate predators that we have long known are important for regulating populations of insect prey in both natural and agricultural ecosystems (e.g. [26,27]) and are therefore expected to exert strong selective pressures on prey.

Jumping spiders are one group of visually guided predators that may have a lot to teach us about aposematism and the functions of prey colour and pattern more generally. As voracious predators, they have been argued to be important drivers of visual defences in several small arthropod prey species (e.g. [28–32]). Yet most of what we know about how they interact with aposematic colour schemes comes from work with long-wavelength colours (i.e. red). Experiments have shown that some species of jumping spiders that feed on a wide variety of prey in the field specifically attend to and avoid red when making prey choice decisions [17,33–35] and that they show heightened attention to and avoidance of red in the presence of aversive odours from chemically defended bugs [36,37]. However, context and natural history matter. For example, *Evarcha culicivora*, a mosquito specialist jumping spider uses a preference for the colour red to identify its preferred prey of blood-carrying mosquitoes [38]. This highlights the fact that not all jumping spider species respond to colours that are typically associated with aposematism in the same way. With more than 6500 species to consider [39], this group provides an opportunity to explore and test broad generalizations about how animals use aposematic colours in nature to decide what to eat and what to avoid.

Another reason that jumping spiders, as a group, may offer rich insights into the study of aposematism is that they do not all see aposematic displays the same way. Most of the studies that consider aposematic displays from a jumping spider's perspective have focused on the colour red (described above), yet recent work has suggested that this ability to see and discriminate red (and other long-wavelength colours) may be limited to only a few small clades of jumping spiders [40,41]. Many species are thought to be dichromats with limited ability to see and discriminate the reds and oranges that are often featured in aposematic displays [40,41]. Such dichromats can still probably recognize the bold high-contrast patterns of dark and light (e.g. black and white stripes, spots, etc.) that are often incorporated into these displays, and they might rely on these achromatic patterns more heavily if they have limited colour vision. Studies of aposematism rarely focus on achromatic patterns alone (without some element of colour) [42].

At least two jumping spider species have been found to attend to such patterns (i.e. black and white stripes) when foraging. In prey choice experiments where *Maevia inclemens* was given the choice between live termites affixed with patterned paper capes, spiders oriented more frequently to termites with striped capes compared with those that had either solid grey or solid yellow-orange capes [43]. This suggested that the striped termites were the most conspicuous or attention-grabbing. However, this increased conspicuousness did not result in an increase in predation as the three types of termites were attacked at equal rates [43]. In similar experiments with *Plexippus paykulli,* responses to striped termites were slightly different [44]. In this species, spiders oriented to striped and unstriped (grey) termites at similar rates, but the striped termites were attacked at significantly lower rates than unstriped termites [44]. Both sets of data suggest that there is something interesting about stripes: either they can attract attention without a corresponding increase in predation or they can effectively deter attacks after they are seen. While these previous studies aimed to assess the responses of spiders to striped prey, they were not designed to disentangle the effects of a prey item's internal striped patterning versus the prey item's contrast with the white background on which they were presented to the spiders.

The goal of the present study was to understand how two additional species of jumping spiders (*Phidippus regius* and *Habronattus trimaculatus*) respond to black and white stripes on prey. We used choice tests with live termites (that had their dorsal patterns manipulated with paper capes similar to those described above) to build on previous work in two ways. First, by asking how these two additional species respond to striped prey, we can begin to assess whether heightened attention to and/or aversion to stripes is likely to be a general feature of jumping spider foraging, and to what extent it varies across species with and without colour vision and with different sensitivities to prey toxins. *Phidippus regius* is a presumed dichromat [41] with a robust ability to withstand prey toxins: while they sometimes avoid chemically defended prey [45,46], they have also been documented to sometimes consume chemically defended prey in the field and laboratory and often suffer only minor consequences from the ingestion of prey toxins [46,47]. By contrast, *H. trimaculatus* has trichromatic vision [41] and a higher sensitivity to unpalatability and toxicity in prey, meaning that they are more easily deterred by unpalatability and suffer more severe consequences if they consume toxic prey [47]. A second way that the present study builds on previous work is that our experiments were designed to disentangle the effect of internal body pattern from contrast with the background. Here, by giving spiders the choice between striped, white and black prey moving on a white background, we could determine whether any effects we observed were the result of the prey contrasting highly with the background (as is the case for both black and striped prey) or solely the result of internal patterning (present only on the striped prey).

Previously published studies on *M. inclemens* and *P. paykulli* (cited above) lead us to predict that stripes on prey would attract attention and/or reduce attacks. However, despite the clear biological differences between the two species we chose for the present study, we had no clear *a priori* predictions about if and how any patterns might differ between the species. On the one hand, we might expect *P. regius,* with its increased ability to withstand prey toxins [47], to be less discriminating when foraging and less likely to be deterred by a common aposematic signal like stripes compared with *H. trimaculatus*. On the other hand, the fact that *P. regius* has a limited ability to see and discriminate long-wavelength colours might mean that they must be more attentive to achromatic warning signals like stripes compared with *H. trimaculatus* that can rely on red and other long-wavelength coloration as well. Only with a nuanced understanding of how different species of jumping spiders respond to common aposematic patterns can we make sense of how jumping spiders fit into and broaden our understanding of how conspicuous and colourful prey defences work.

## 2. Methods

We collected spiders of both species (32 *P. regius*: all immatures; 46 *H. trimaculatus*: 35 immatures, seven adult females and four adult males at testing time) from areas around north central Florida between September and December in 2015 and 2016. All were housed in individual clear plastic boxes (4 × 4 × 5–1/16 cm) with an artificial green plant to provide enrichment [48]. The spiders were housed under standard laboratory lighting on a 12 : 12 light : dark cycle. We fed spiders three times per week with juvenile crickets (*Gryllodes sigillatus*) in an amount that was approximately equivalent to the spider's own body size and sprayed their boxes twice per week with water. The time between collection and testing varied from between 1 and 163 days. We did not feed the spiders on the day prior to their prey choice tests; we chose this feeding protocol in an effort to increase their motivation to participate, while not making them so hungry that they would attack prey indiscriminately.

A separate group of laboratory-reared *P*. *regius* (*n* = 30) were tested in addition to the field-collected spiders described above. These spiders were the progeny of five different mothers; no more than seven individuals from the same mother were included in our study. Because these spiders were raised only on crickets (as described above) they never had any prior experience with striped prey. As such, any responses to stripes seen in this group are likely to be the result of innate biases. Each test spider was used only once.

Adults of these two species differ substantially in adult body size. Adult female *P. regius* are approximately 14.8 mm [49] while adult female *H. trimaculatus* are approximately 6.9 mm [50] in total body length. However, because our sample included spiders that were mostly juveniles at testing time, members of the two species were similar in total body length with *P. regius* only slightly larger (*P. regius* ranged from 3 to 9 mm, *H. trimaculatus* ranged from 2 to 6 mm; *t*-test, *t*_105_ = 1.92, *p* = 0.057, *n* = 107; note that one spider was missing size data) but this difference was not statistically significant.

### 2.1. Manipulation of prey pattern

**Figure 1.**
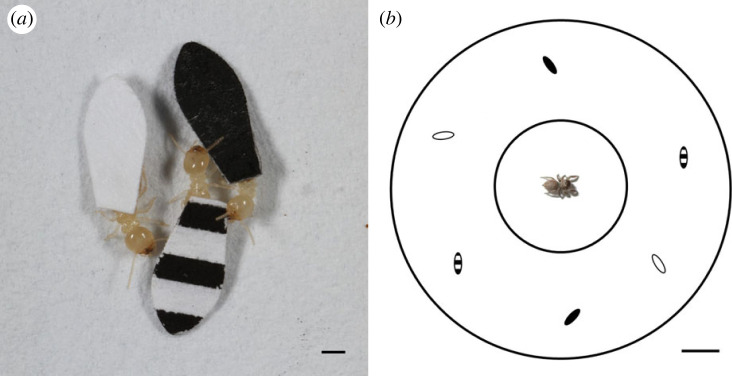
Methods for manipulating dorsal patterns in Eastern subterranean termites (*Reticulitermes flavipes*) and presenting them to jumping spider predators (*Phidippus regius* and *Habronattus trimaculatus*) in prey choice experiments. (*a*) Termites affixed with patterned paper capes. Scale bar represents 1 mm. (*b*) Schematic of the experimental set-up. We allowed the spiders to acclimatize in the lidded central chamber before releasing them into the rest of the arena to prey on the termites. Scale bar represents 1 cm.

For prey choice tests, we used field-collected worker termites (*Reticulitermes flavipes*) collected from natural populations in Gainesville, FL. These termites are common in the same habitats as the two jumping spider species studied. Both spider species are generalist predators that will readily attack most insects that they encounter in the field, including termites (L.A.T. 2013–2023, personal observations). To alter the dorsal patterns of these termites, we affixed small pieces of paper to the thorax using non-toxic Elmer's glue (Elmer's Product, Inc., High Point, NC, USA) and aligned these ‘capes' to cover the abdomen (figure 1). These paper capes were similar in surface area (approx. 5 mm long and 2 mm wide) to the wings of winged, alate termites of the same species, so the termites were able to easily and naturally move while wearing them. We used an HP LaserJet 1020 (Hewlett-Packard, Palo Alto, CA, USA) and standard 20 lb. bond white printer paper (item 420283, Office Depot, ODP Corporation, Boca Raton, FL, USA) with ink printed on both sides to make capes of three different treatments: black, white and striped. The striped capes had a white background with three horizontal black stripes (figure 1). This method of termite ‘caping' has been used successfully in several previously published studies of jumping spider foraging [43,44,51]. To ensure that the differently patterned capes did not differentially affect termite behaviour, we ran pilot tests where we caped 15 termites of each type (black, white, striped) and placed them individually in empty paper-lined 9 cm Petri dishes (the same set-up as used below in our prey choice tests). We observed each termite for 2 min and used stopwatches to record the total time they spent actively moving. If they stopped moving for more than 1 s, the stopwatch was stopped until they started moving again. We also recorded movement rates of 15 termites with no capes for comparison.

### 2.2. Prey choice tests

We conducted prey choice tests within 9 cm Petri dishes lined with white filter paper. The paper provided a consistent visual background and a substrate on which the termites could grip and move around naturally. At the start of each test, we placed six termites (two white, two black, two striped) onto the floor of the paper-lined dish (see electronic supplementary material, video). The rationale for using six termites per trial was that while we could not control the behaviour of the individual termites, this number increased the chances that the test spider would view multiple prey items before making an attack. This termite dish was placed in a larger opaque white plastic container to minimize visual disturbance. We then placed a test spider into a smaller (3.5 cm) lidded Petri dish in the centre of the 9 cm dish. We gave the spider 10 min to acclimatize in this smaller dish; during this time the spider was able to view the termites wandering around it and see that there were several choices before being given the opportunity to attack. After the acclimatization period, the lid of the small Petri dish was removed, releasing the spider into the larger termite dish (figure 1). We gave the spider 5 min to exit the small Petri dish; if there was no exit within this time (this occurred in seven cases, all *H. trimaculatus*), the trial ended and the spider was retested on the following testing day. After the spider exited the dish, it had another 10 min to capture its first prey item. The trial ended as soon as a termite was captured or when the 10 min mark was reached. After each trial, all dishes were cleaned with ethanol and allowed to dry fully. Each termite was used in only one trial. During trials (starting from when the spider was first introduced into the acclimatization dish), we recorded the treatment of termite that the spider first oriented to. This orientation response is clear and unambiguous in foraging jumping spiders; when something gets their attention, they quickly swivel their bodies so that the object of interest is in the visual field of their large forward-facing principal eyes [52] before deciding whether to attack it. We also recorded the treatment of any termites attacked during the trials.

For the *P. regius* trials, we also recorded where on the body the spider attacked (head, side or back). (See rationale below in the Statistical analyses section.)

None of the trials were video-recorded; we collected all data in real time.

### 2.3. Statistical analyses

#### 2.3.1. Effect of capes on termite movement

We used a *t*-test to assess whether caped termites moved at different rates than uncaped termites and ANOVA to assess whether there were any differences between the three cape treatments.

#### 2.3.2. Effect of termite treatment on spider orientation

For each of the two spider species, we used likelihood ratio *χ*^2^-tests to ask whether the spiders were more likely to orient to some treatments over others, testing our *a priori* prediction that stripes would be quicker than the other treatments to capture a spider's attention. If they did not orient to all three treatments equally (i.e. if the *χ*^2^-test showed that there were significant differences among groups), we went on to use 95% confidence intervals (i.e. score confidence intervals for categorical data) to assess the pairwise differences in orientation frequencies among the three different termite treatments.

#### 2.3.3. Effect of termite treatment on spider attack

Our approach for analysing the attack data was identical to that described above for the orientation data. For each species, we used likelihood ratio *χ*^2^-tests to ask whether the spiders were more likely to attack some treatments over others, testing our *a priori* prediction that spiders would have the lowest attack rates on striped prey. If they did not attack all three treatments equally, we used 95% confidence intervals to assess pairwise differences in attack frequencies among the three different termite treatments.

#### 2.3.4. Exploratory analyses

In addition to the main tests of our *a priori* predictions described above, we ran several exploratory analyses to better understand the patterns in our data.

First, for each species, we used likelihood ratio *χ*^2^-tests to ask whether the treatment of the termite that a spider first oriented to affected the treatment of the termite that the spider ultimately attacked and whether spiders were more likely than chance to attack a termite of the same treatment as the one to which they first oriented. The rationale for this test was that we wanted to understand if a spider's decision to attack a termite might be indirectly influenced by whether or not that termite was a conspicuous treatment that got the spider's attention first.

Second, in light of qualitatively similar results for the two jumping spider species but different patterns of statistical significance (probably driven in part by different sample sizes; see Results) we analysed the data from the two species together in a single model to ask if they differed from each other in their responses. The goal of this analysis was to help us understand whether there was evidence that the two species were truly responding differently from one another, as opposed to an alternative scenario where the patterns for the two species were similar, with only one crossing the threshold to reach statistical significance. Specifically, we used likelihood ratio *χ*^2^-tests to assess whether the two species differed in the treatment of the termites to which they first oriented and the treatment of termites that the spiders ultimately attacked.

Third, we used likelihood ratio *χ*^2^-tests to ask whether the termite treatments affected which part of the body the *P. regius* spiders attacked (at the head, from the side, or from behind). The rationale for this test was that we already know that *P. regius* attacks flies and caterpillars from different directions, presumably increasing their capture success [53]. When it comes to aposematic prey, the pattern of a prey item might be used by a spider to assess the risk of danger; as such, spiders might attack patterns associated with danger (e.g. those typically associated with aposematism like stripes) from a safer orientation, such as from the rear or the side rather than the head.

Finally, because our *P. regius* test spiders included both field-collected and laboratory-raised individuals (unlike our *H. trimaculatus* spiders that were all field-collected), we used likelihood ratio *χ*^2^-tests to ask whether these two types of *P. regius* (field-collected versus laboratory reared) differed in the treatments of termites that they first oriented to or that they attacked. The rationale for this test was to gain insight into whether the patterns that we see in our data are most likely the result of innate or learned biases (or some combination). For example, if the field-collected spiders were biased against attacking stripes, but the naive laboratory-raised spiders were not, it might suggest that biases against stripes were learned from experience in the field.

All data were analysed using JMP Pro 17.0.0. Raw data are archived on DRYAD and are available here: https://doi.org/10.5061/dryad.m37pvmd7d [54].

## 3. Results

### 3.1. Effect of capes on termite movement

Termites wearing capes did not move at different rates compared with uncaped (unmanipulated) termites (mean movement *t*_58_ = 0.15, *p* = 0.88). There were no differences in movement rates among termites with the three cape treatments (*F*_2,42_ = 0.70, *p* = 0.50).

All individuals of *P. regius* both oriented towards and attacked at least one termite during our trials. All but one of the *H. trimaculatus* individuals oriented towards at least one termite, but only 25 out of 46 (54%) attacked a termite within the allotted time. As a result of this difference in voracity, our sample sizes for the attack data are substantially smaller for *H. trimaculatus* (25 attacks) compared with *P. regius* (62 attacks).

### 3.2. Effect of termite treatment on spider orientation

**Figure 2.**
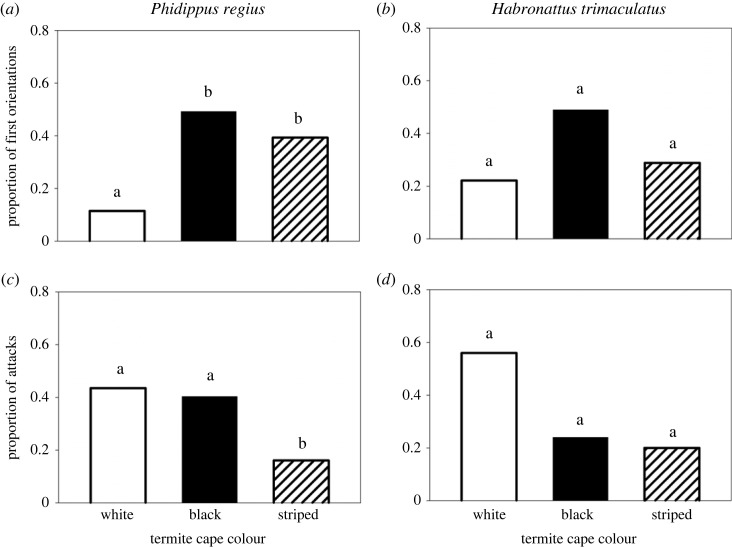
Results of prey choice tests with live termites using two jumping spider species as predators (*Phidippus regius* and *Habronattus trimaculatus*). Rates of orientation to the three different termite treatments by (*a*) *P. regius* and (*b*) *H. trimaculatus.* Rates of attack on the three different termite treatments by (*c*) *P. regius* and (*d*) *H. trimaculatus*. Different letters above the bars indicate significant differences.

*Phidippus regius* did not direct their first orientations equally across the three termite types (likelihood ratio *χ*^2^ = 16.37, *p* = 0.003, *n* = 61; figure 2*a*; note that orientation data are missing for one spider due to a termite pile-up that made it impossible to tell which termite it first oriented to). The spiders directed their first orientations most often to black (*n* = 30 (49%)) and striped (*n* = 24 (39%)) termites, and least often to white (*n* = 7 (11%)). Assessment of the 95% confidence intervals suggests that the rate of orientation to black and striped were statistically similar (i.e. the confidence intervals (CI) overlap: black = 0.371–0.614, striped = 0.281–0.519), but that the spiders oriented to white termites significantly less often than either black or striped (CI for white: 0.057–0.218; figure 2*a*).

For *H. trimaculatus*, we found that there were no statistically significant differences in the termite types that they first oriented to (likelihood ratio *χ*^2^ = 5.02, *p* = 0.081, *n* = 45; white: *n* = 10 (22%), CI = 0.125–0.363; black: *n* = 22 (49%), CI = 0.351–0.630; striped: *n* = 13 (29%), CI = 0.177–0.434; figure 2*b*).

### 3.3. Effect of termite treatment on spider attack

For *P. regius*, despite the finding that black and striped termites were more likely to capture attention (i.e. more likely to elicit orientations; see above), we did not find that these treatments elicited more attacks. There were differences between the three termite groups in how often they were attacked (likelihood ratio *χ*^2^ = 9.43, *p* = 0.0089, *n* = 62; figure 2*c*). Examination of the 95% confidence intervals suggests that striped termites were attacked at levels that were significantly lower than either black or white (striped: *n* = 10 (16%), CI = 0.090–0.272, figure 2*c*). White termites and black termites were attacked at similar rates (white: *n* = 27 (44%), CI = 0.319–0.559; black: *n* = 25 (40%), CI = 0.290–0.527; figure 2*c*).

For *H. trimaculatus*, there were no statistically significant differences in attack rates between the three termite types (likelihood ratio *χ*^2^ = 5.48, *p* = 0.065, *n* = 25; white: *n* = 14 (56%), CI = 0.371–0.733; black: *n* = 6 (24%), CI = 0.115–0.434; striped: *n* = 5 (20%), CI = 0.089–0.391, figure 2*d*).

### 3.4. Exploratory analyses

For *P. regius*, the termite treatment that they first oriented to was related to the treatment that they went on to attack (likelihood ratio *χ*^2^ = 6.51, *p* = 0.011, *n* = 61), but this pattern was driven by the individuals that first oriented to either black or white and then went on to attack the same colour (table 1). Spiders that oriented first to striped termites were most likely to go on to attack white (table 1). By contrast, for *H. trimaculatus*, there was no relationship between the treatment first oriented to and what the spiders went on to attack (likelihood ratio *χ*^2^ = 1.99, *p* = 0.159, *n* = 25; table 1).

**Table 1.** Relationship between the treatment of the termite that a spider first oriented to and the treatment of the termite that the spider ultimately attacked in prey choice experiments with *P. regius* and *H. trimaculatus*.

treatment of termite first oriented to	attacked white	attacked black	attacked striped
*Phidippus regius*			
white	6 (86%)	1 (14%)	0 (0%)
black	8 (26%)	18 (60%)	4 (13%)
striped	12 (50%)	6 (25%)	6 (25%)
*Habronattus trimaculatus*			
white	4 (67%)	2 (33%)	0 (0%)
black	3 (27%)	4 (36%)	4 (36%)
striped	7 (88%)	0 (0%)	1 (13%)

When pooling the data for the two species together, we found no differences between the species in which treatments they oriented to first (likelihood ratio *χ*^2^ = 2.66, *p* = 0.26, *n* = 106) or which treatments they attacked (likelihood ratio *χ*^2^ = 2.16, *p* = 0.34, *n* = 87).

In an exploratory analysis for the data from *P. regius*, we asked whether spiders attacked termites at different areas of the body depending on their treatment. While the spiders overwhelmingly attacked the termites from the head end (rather than from the back or side, likelihood ratio *χ*^2^ = 71.53, *p* < 0.0001, *n* = 61), this attack direction did not vary depending on the treatment of the termite (likelihood ratio *χ*^2^ = 3.87, *p* = 0.42, *n* = 61).

When we compared field-caught and laboratory-reared *Phidippus regius*, we found no difference in the treatment of the first termite oriented to (likelihood ratio *χ*^2^ = 0.83, *p* = 0.66, *n* = 61) or the treatment of the termite first attacked (likelihood ratio *χ*^2^ = 0.71, *p* = 0.70, *n* = 62).

## 4. Discussion

Our results combined with previously published studies testing for responses to similar types of striped prey [43,44] collectively suggest that stripes are salient to foraging jumping spiders from across the salticid phylogeny. While the subtleties vary by species, stripes had the effect of either increasing attention to a prey item or reducing the likelihood of attack (and sometimes both).

As a particularly voracious species, *P. regius* gave us the largest opportunity to detect a clear effect in our experiments as these spiders (*n* = 62) attacked a termite in every trial. We found that both black and striped termites captured the attention of *P. regius* sooner than white termites. From this, we can conclude that high achromatic contrast is probably what is driving the effect in this species, whether it is the internal contrast of the black and white body stripes, or the contrast of the solid black termite on the white background. Given the jumping spider visual system, this might not be surprising that contrast alone rather than a detailed pattern is enough for triggering an orientation response. Jumping spiders have eight eyes, with the large forward-facing principal eyes being the ones that are renowned for their ability to detect fine details (reviewed in [55,56]). However, the orientation response is triggered by the much smaller motion-detecting secondary eyes positioned on the sides of the spider's head; these eyes are sensitive to movement of objects that contrast with their background allowing a spider to reorient its body to place an object of interest in the visual field where it can be observed more carefully by the principal eyes [57].

When assessing attack rates in *P. regius*, the striped termites were attacked less often than either solid black or solid white. This suggests that once the spider has oriented to the prey and scanned it with its high acuity principal eyes, it is the internal body patterning, specifically, that triggers aversion, and not just the prey item's contrast with the background. More work should be done to better understand whether certain internal body patterns on aposematic prey are more or less effective at deterring predation (e.g. stripes versus spots, see [42,58]) and whether the degree of achromatic contrast present within a pattern matters [42]. We focused our study on high-contrast stripes as a starting point for understanding aposematism more broadly. We might expect patterns of stripes and lines to be particularly salient to jumping spiders compared with other aposematic patterns because of their relevance in other contexts. Specifically, early behavioural work with jumping spiders ([59]; reviewed by Land [55]) highlighted the importance of dark black lines in object discrimination. Drees [59] showed that an image of a black dot on a moving paper card was readily perceived as prey and attacked, but if that same dot had pairs of oblique lines on either side of it (subtly resembling legs), then it was no longer categorized as prey but instead categorized as another jumping spider and males courted it rather than attacking. Similarly, black lines on the wings of some species of metalmark moths and tephritid fruit flies are thought to exploit this same recognition template; when these potential prey items encounter a jumping spider, they display their striped wings and jumping spiders typically respond to them as if they are another jumping spider, waving their own legs at them rather than attacking [30,31,60]. Collectively, this work should remind us that we cannot fully appreciate a predator's response to an aposematic signal like stripes without also considering how the brain makes sense of similar patterns of stripes and lines in other contexts.

While our goal was to understand responses to stripes in the context of aposematism, there are other ways that stripes have been shown to reduce predation in animals, and it is important to consider whether the patterns observed here might be explained by these other (non-mutually exclusive) mechanisms. For example, some animals use stripes as disruptive coloration, which is a form of camouflage that uses highly contrasting pattern elements like stripes to break up an animal's outline, hindering detection by predators (reviewed in [61]). This seems unlikely to explain the findings in our study because this mechanism predicts that we would have seen the lowest orientation rates to striped prey, which is contrary to what we found. Another way that animals use stripes to avoid predation is by a phenomenon called motion dazzle, where stripes or other pattern elements impair a predator's ability to assess prey speed and trajectory, thereby inhibiting capture, particularly in fast-moving prey [62,63]. This too seems unlikely to explain our findings, as this phenomenon would predict that we would see the highest number of missed attacks on striped prey compared with the other treatments. In revisiting our trial notes, we observed three missed attacks across our 108 trials. All of these occurred in tests with *P. regius* and none were on striped termites (two were on black and one was on white). While these two phenomena are unlikely to explain our findings here with caped termites on a white background, it would be interesting to know whether either of these mechanisms, or others, are relevant to jumping spider predators that are hunting for striped prey in the field on natural backgrounds.

Our exploratory analyses offer support for the idea that the patterns observed in *P. regius* are at least partially innate, as field-collected spiders (*n* = 36) and laboratory-raised spiders that had never previously experienced striped prey (*n* = 30) did not show statistically different patterns of attention to and aversion to stripes. However, we know that spiders in the genus *Phidippus* can learn aversions from experience with aposematic prey such as milkweed bugs [45,64] and flashing fireflies [29]. As such, it is likely that any decisions they make in the field result from a combination of both innate and learned biases. Both innate and learned biases seem to contribute to aversions to other aposematic signals (specifically, the colour red) in other jumping spider species [17,33–35].

Our results from tests with *H. trimaculatus* were more ambiguous than those from *P. regius*. Despite both species being fed the same diet in the laboratory (with each individual's food quantity being scaled to their body size), and similar-sized specimens of the two species being tested (see Methods), the *H. trimaculatus* were less likely to attack termites within the allotted time in our trials, leading to a substantially smaller sample size of attacks (*n* = 25). In our experience with these two species in the laboratory, this is typical, with *P. regius* being generally more voracious than *H. trimaculatus* (L.A.T. 2013–2023, personal observation). The patterns in the data for *H. trimaculatus* shared some similarities with *P. regius* (figure 2), except that striped and black termites were attacked at similar rates (in contrast with a lower attack rate on stripes by *P. regius*). For *H. trimaculatus,* the differences between the three termite types did not meet the threshold for statistical significance for either the orientation data or the attack data (see Results). When reporting negative results such as these, it is important to consider the possibility that the lack of significance is due to small sample size, which may be the case here. To allow readers to assess this possibility, we included proportions and 95% confidence intervals alongside our results [65,66]. The idea that the patterns in the data for the two species share some similarities is supported by our exploratory analysis showing that the species did not differ statistically from one another in the termite treatment first oriented to or the termite treatment first attacked (see Results); yet, again, one could argue that the smaller sample size for *H. trimaculatus* could be contributing to this lack of a statistical difference.

Our exploratory analyses might tell us something about how the aversion to stripes played out during our trials, and how it might play out in real settings when spiders (or other predators) are given the opportunity to choose among several living prey items moving around them. In *P. regius*, the termite treatment that an individual first oriented to during their acclimatization period (when they were enclosed in a clear acclimatization chamber) predicted the treatment that they went on to attack when they were released. However, this pattern only held up when the spiders first oriented to either white or black termites. If the first termite they oriented to was striped, they were twice as likely to attack a white termite than either striped or black. We can speculate that seeing stripes on a novel prey item may trigger a heightened level of caution, causing the spiders to view their choices more carefully, and to ultimately choose the most inconspicuous option (white). When either of the other choices (white or black) were viewed first, they may have simply prioritized tracking the same individual that first captured their attention, even with the other termites moving around them. While these same statistical patterns did not hold for *H. trimaculatus* (perhaps due again to the smaller sample size), the patterns in the data were qualitatively similar: of the eight spiders that first oriented to striped termites, seven of them went on to attack white termites (table 1). Our experiment capitalized on the use of natural, freely moving live prey that spiders could physically approach and capture, but this also meant that we could not control the natural variation among individual prey items. It might be fruitful to complement this work with additional techniques—for example, by using recently refined jumping spider eye-trackers that allow researchers to observe the moving gaze of spiders as they watch highly controlled video-projected prey [67,68].

If seeing stripes on a novel prey item (i.e. a termite wearing a striped cape) makes these spiders proceed more cautiously, as we speculate above, we might have expected striped termites to be attacked from different orientations (perhaps ‘safer' orientations) compared with non-striped ones. However, it is difficult to say what the safest way to attack a novel aposematic prey item might be, as any risk is likely to vary by the specific nature of the prey species' defence (for example, the specific location on the body where chemical defences are released [69]). In our analysis of attack direction in *P. regius*, we found that attacks were overwhelmingly (85%) targeted at the head end of our caped termites, regardless of the termite's treatment; this might simply reflect a general strategy for attacking unfamiliar, flightless prey. To put this in the context of how other generalist jumping spiders hunt, we see many examples of head-on attacks when confronting undefended prey (e.g. thrips and caterpillars [53,70], virtual caterpillar-shaped prey with features of flies and thrips [71], and the false heads of hairstreak butterflies [32]). But undefended prey items are also sometimes targeted indiscriminately (without a bias in attack direction), particularly when they are relatively small and have a high probability of escape (e.g. flies and hoppers: [53,72–74]). When it comes to attacking more dangerous prey, we find examples of predatory specialists that strategically attack head-on; for example, some ant specialist jumping spiders consistently attack from the head end and grasp the ant's thorax in a way that avoids both bites and stings (e.g. [75,76]). We also find examples of specialists that strategically avoid the head, as is the case in some *Portia* jumping spiders that detour to avoid the defensive spit of spitting spiders [77]. There has been considerably less work examining the specific attack strategies used by generalist jumping spiders when attacking aposematic prey, but at least one study reported anecdotal observations of *Phidippus* jumping spiders attacking milkweed bugs from the head end before going on to reject them [45]. While our analysis of attack direction here was exploratory, and not a focus of our study, it would be interesting for future work to further examine if and how jumping spiders take aposematic colour schemes into account when making decisions about how to approach and attack prey.

With data now from four jumping spider species attacking patterned caped termites, we can begin to speculate that heightened attention to stripes and/or increased aversion to stripes may be general features of jumping spider foraging, with subtle differences between species. These species come from across the salticid phylogeny and include members from two major clades (Saltafresia and Marpisoida) and four distinct subtribes [78]. They also include at least one known trichromat (*H. trimaculatus:* [41]), one presumed dichromat (*P. regius*: [41,79]), and two species for which visual capabilities are less well known (*M. inclemens* and *P. paykulli*, see [80,81]). The fact that stripes seem to be salient to all of them, even if in subtly different ways, may help explain why so many small insect prey incorporate stripes into their aposematic displays. Some notable examples that co-occur with our study species include the smallest instars of black and white striped caterpillars (e.g. monarchs: [82]), treehopper nymphs (e.g. *Platycotis vittata* and *Umbonia crassicornis* [83,84], cucumber beetles [85] and the boldly striped eggs and nymphs of harlequin bugs (*Murgantia histrionica* [86]). These tiny prey items are probably too small to be of much interest to most of the better studied larger vertebrate predators (e.g. birds), yet their small size probably makes them excellent targets for most salticids. While some of these examples of insect prey include long-wavelength colours in addition to dark and light stripes, those long-wavelength colours are unlikely to be particularly conspicuous to salticids without colour vision. This highlights a major benefit of high-contrast achromatic stripes in aposematic displays: they should be conspicuous to all salticids, including di-, tri- or tetra-chromats (as well as other visual predators with and without colour vision). They also allow for the addition of long-wavelength colours, if these colours provide added benefits for the subset of predators that can see them. Indeed, evidence from birds with excellent colour vision suggests that achromatic patterns and long-wavelength colours may play different roles in defending aposematic prey (e.g. [87–89]).

The fact that there seems to be subtle variation in responses to striped prey across salticids makes this group particularly well suited for phylogenetic comparative studies that test broad hypotheses about how and why predators respond to different components of aposematic displays. For example, an interesting next step would be to replicate the tests done here across a phylogenetic scale that includes multiple closely related species pairs with and without colour vision [40] and with different sensitivities to prey toxins [47]. This would allow us to test ideas about how a species' degree of colour vision (and ability to rely on long-wavelength warning colours like red and orange) and the relative costs of ingesting prey toxins might influence their attention to and avoidance of aposematic patterns that contain only achromatic (black and white) elements. If species only have dichromatic vision (like *P. regius*), we might expect them to rely more heavily on achromatic cues compared with trichromats (like *H. trimaculatus*) that can also rely on reds and oranges in warning displays. And species that are most susceptible to prey toxins might be more attentive to aposematic colour schemes broadly compared with those that can ingest prey toxins with little cost [47]. Because of their multiple evolutionary origins of tri- and tetra-chromacy [40,41], the salticids offer a unique opportunity to test such ideas that is not possible in many other taxa [90]. Most other work on aposematism, including work that focuses specifically on achromatic cues (e.g. [42,91]) has been done with animals that have good colour vision. Yet natural predator communities typically include taxa with diverse visual systems with and without colour vision (reviewed in [56]) as well as species that have very different sensitivities to prey toxins [15,92]. Understanding how these two axes of variation affect responses to aposematic signals should provide insight that is broadly relevant across animal groups.

Might our results presented here also help us understand conspicuous signals sent outside of the context of aposematism? One feature shared by all four jumping spider species tested with striped prey is that they are sexually dichromatic, with males displaying bold patterns of black and white to females during courtship (electronic supplementary material, figure S1). This is no accident—our interest in understanding how stripes affect foraging decisions was initiated by a larger effort to understand why male jumping spiders from across the salticid phylogeny so often incorporate stripes into their courtship displays. We have hypothesized elsewhere that if stripes are particularly salient to females in the context of foraging, then a pre-existing attentiveness to stripes may make them well suited for capturing and maintaining a female's attention during courtship [43,44]. In addition to capturing female attention, a second challenge for male jumping spiders is avoiding sexual cannibalism (e.g. [44,49,93]). If females have an aversion to attacking striped prey (as our data here show), then males might benefit from striped display patterns that exploit this female predatory aversion, reducing the chances that a female will attack the courting male (see broad literature on ‘sensory traps', [94]). A similar idea has been proposed by Land [55], who suggested that striped body patterns in males might exploit the attention that female jumping spiders pay to black lines (i.e. the presence of multiple legs) when deciding whether a suitor is another spider or a prey item. Studies with *M. inclemens* and *P. paykulli* have provided mixed support for these ideas to date. Striped patterning on males does not appear to reduce cannibalism risk during courtship interactions in either *M. inclemens* [95] or *P. paykulli* [44]. But stripes do increase mating success in male *P. paykulli* [44]*.* More work is needed to assess whether female attentiveness to stripes during foraging and courtship are related*.* Here again, a phylogenetic comparative study could provide broad insight by asking whether a species' use of stripes in male courtship displays is correlated with the strength of a female's attention to and/or avoidance of stripes in foraging.

## Data Availability

Data are available from the Dryad Digital Repository: https://doi.org/10.5061/dryad.m37pvmd7d [54]. Figure S1 and a video showing the movement of caped termites in our test arenas are provided in electronic supplementary material [96].
